# How to promote usage of telehealth interventions for farmers' mental health? A qualitative study on supporting and hindering aspects for acceptance and satisfaction with a personalized telephone coaching for depression prevention

**DOI:** 10.1016/j.invent.2023.100671

**Published:** 2023-09-19

**Authors:** Janika Thielecke, Claudia Buntrock, Johanna Freund, Lina Braun, David D. Ebert, Matthias Berking, Harald Baumeister, Ingrid Titzler

**Affiliations:** a Department of Clinical Psychology and Psychotherapy, Friedrich-Alexander-Universität Erlangen-Nürnberg, Erlangen, Germany; b Department of Sport and Health Sciences, Technical University of Munich, Munich, Germany; c Institute of Social Medicine and Health Systems Research, Otto von Guericke University Magdeburg, Magdeburg, Germany; d Department of Clinical Psychology and Psychotherapy, Ulm University, Ulm, Germany

**Keywords:** Interviews, Acceptance, Satisfaction, Telephone coaching, Depression, Farmers

## Abstract

Low-threshold and remotely delivered preventive interventions, like telephone coaching, are warranted for farmers who experience multiple risk factors for depression, live in underserved areas, and show low help-seeking behavior. Factors facilitating uptake and actual use of effective remote interventions are important to reduce depression disease burden. This study aimed at identifying factors that potentially can influence acceptance of and satisfaction with a telephone coaching in this occupational group.

Semi-structured interviews were based on the ‘Unified Theory of Acceptance and Use of Technology’, the ‘Evaluation’, and ‘Discrepancy’ models for satisfaction. Interviews were conducted with 20 of 66 invited participants of a 6-months telephone coaching during an effectiveness or implementation study. Audio-recorded interviews were transcribed and analyzed (deductive-inductive qualitative content analysis). Independent coding by two persons resulted in good agreement (Κ = 0.80). Participants validated results via questionnaire.

Overall, 32 supporting (SF) and 14 hindering factors (HF) for acceptance and satisfaction were identified and organized into five dimensions: Coaching result (SF = 9, HF = 3), coach (SF = 9, HF = 1), organization (SF = 5, HF = 2), the telephone as communication medium (SF = 4, HF = 5) and participant characteristics (SF = 5, HF = 3). Most named SFs were ‘Flexible appointment arrangement’ (n = 19/95 %) and ‘low effort’ (n = 17/85 %), while most reported HFs were ‘lack of visual cues’ (n = 12/60 %) and ‘social/professional involvement restricts change process’ (n = 10/50 %).

The perceived changes initiated by coaching, a low effort through telephone conduct, and the indicated personalization were identified as important influencing factors on acceptance and satisfaction based on interviewees' statements. Both may be further enhanced by offering choice and advice for delivery formats (e.g., video-calls) and training of coaches in farm-related issues.

**Study registration:**

German Clinical Trial Registrations: DRKS00017078 and DRKS00015655.

## Highlights

• The interview study provides insights on factors influencing acceptance and satisfaction with a telephone coaching for farmers
• The telephone coaching is personalized in topics on individual participants needs and in length and frequency
• Influential factors included the coaching result, coach, organization, participant characteristics and the delivery medium
• Interviewees’ statements suggest that intervention results, low-threshold availability and personalization are important
• Study results can be used to further develop and adapt the intervention and promote uptake and use in routine care

## 1 Introduction

Major depression disorder (MDD^1^) is a highly prevalent mental disorder (Gutiérrez-Rojas et al., 2020) and ranks first as single contributor to all years lost to disabilities worldwide (World Health Organization, 2017). Effective psychological and pharmacological treatments exist, but only one out of five individuals with MDD receives adequate care (Bromet et al., 2018). Preventive interventions could target individuals at risk for depression and improve mental health care in an early stage.

Psychological interventions for the prevention of MDD have been shown to effectively reduce depression symptoms (Cuijpers et al., 2014) and avoid or at least delay MDD onset (Cuijpers et al., 2021; van Zoonen et al., 2014) in people with known risk factors (selective prevention) or with subthreshold symptoms (indicated prevention). Nevertheless, uptake of indicated preventive interventions is low with estimations that less than one in a hundred individuals with subthreshold depression begins a preventive program (Cuijpers et al., 2010).

A promising approach to address the challenge of low uptake of mental health services could be telehealth interventions which can be used flexibly in time and location and thus, facilitate health care access (Ebert et al., 2018; Snoswell et al., 2021). Easily accessible preventive services are especially warranted for farmers and related professions, who experience multiple risk factors for depression (e.g., long working hours, uncontrollable weather conditions) (Daghagh Yazd et al., 2019) while often living in remote, underserved areas (Myers, 2019; Salize et al., 2007; Weinhold and Gurtner, 2014) and showing low help-seeking behavior for mental health problems (Dollman et al., 2021; Judd et al., 2006; Wrigley et al., 2005).

The nationwide pilot project ‘With us in balance’ by the German Social Insurance for Agriculture, Forestry and Horticulture (SVLFG^2^) is therefore addressing this target group by implementing internet- and tele-based interventions for the indicated prevention of depression. Two randomized controlled trials (RCTs)^3^ have shown that these guided and tailored internet-based interventions (Braun et al., 2021a, Braun et al., 2021b, Braun et al., 2019) and the personalized telephone coaching (Thielecke et al., 2022, 2020) can effectively reduce depressive symptom severity and promote mental health in farmers when compared to a control group receiving psychoeducational material. Actual uptake and use by the target population are evaluated in the implementation study ImplementIT^4^ (Freund et al., 2020). The mixed-methods study ImplementIT includes the views of various stakeholders to adequately address the needs of the population, further develop the intervention and promote its use (Damschroder et al., 2009; Proctor et al., 2011). For the perspective of participating farmers, the pre-specified research focus was on aspects related to acceptance of and satisfaction with the intervention.

Acceptance, describing attitudes toward and perception of a service or product and their relation to its adaptation (Venkatesh et al., 2003), has been shown to influence actual uptake of new digital interventions, both in industry (Venkatesh et al., 2003) and in (mental) health care (Harst et al., 2019; Philippi et al., 2021; Sora et al., 2021) settings. Participants' satisfaction – i.e., the subjective evaluation of multiple facets of an experienced health care service with regard to a-priori expectations (Ware et al., 1983) – is an important criterion for health care quality and supports the development of health services (Samartzis and Talias, 2020). For telehealth intervention in patients with mostly somatic conditions, a recent review identified treatment effectiveness, preference, and ease of use as most important contributing factors to satisfaction (Kruse et al., 2017). Reported patient experiences with telephone-delivered cognitive behavioral therapy (CBT^5^) for MDD highlight overall acceptance of the intervention, the convenience of remote delivery, and a good therapeutic relationship (Bee et al., 2010; Haller et al., 2019). Participants in these studies also report an initial doubt about the effectiveness, technical problems, and lack of visual feedback as disadvantages of telephone-administered CBT. To the best of our knowledge, there is no research on users' experiences with preventive telephone interventions. Qualitative studies report therapeutic guidance, target group adaptation, and flexible use as important factors for intervention use for other remotely delivered health services, namely internet-based interventions for depression (Freund et al., 2022; Holst et al., 2017; Mayer et al., 2019). Low internet skills, lack of individual fit, and data security concerns, are on the other hand described as barriers for intervention use (Braun et al., 2022; Freund et al., 2022; Gerhards et al., 2011; Holst et al., 2017; Mayer et al., 2019). When delivering mental health interventions over telephone, some of these factors could be enhanced (e.g., intense guidance) or circumvented (e.g., need for internet skills), but research is missing.

This study addresses the following research question: ‘Which factors influence the acceptance of and satisfaction with a personalized telephone coaching in farmers?’ in order to best meet the needs of the German farming population with a preventive telephone coaching and optimize uptake in routine care.

## 2 Material and methods

### 2.1 Study design and setting

The study was designed as a qualitative interview study with farmers who took part in a personalized telephone coaching as part of the ‘With us in balance’ project. The experiences of the participants were summarized and structured by employing a qualitative content analysis (Mayring, 2015; Schreier, 2014), as this approach is characterized by the use of highly systematically and structuring steps and the reliance of a strong theoretic foundation. In this study, the theoretical components of the satisfaction and acceptance models were used to assess, describe, and structure the participants experiences with coaching in a phenomenological and pragmatic approach (Ramanadhan et al., 2021). The deductively-inductively identified themes are interpreted to describe aspects potentially influencing acceptance and satisfaction in order to derive recommendations for the ongoing implementation and further intervention development of this new preventive offer for the farming community in Germany.

The ethics committee of the Friedrich-Alexander-Universität Erlangen-Nürnberg had approved this qualitative interview study as part of the implementation study ImplementIT and was registered in the German Clinical Trial Registration (DRKS00017078). Interviewees participated in the telephone coaching as clients under routine care conditions during the pilot phase of ImplementIT or as study participants in the associated RCT (TEC-A,^6^ trial registration: DRKS00015655). Initial recruitment for the underlying studies and the coaching offer was done mostly via personal invitation letter by the SVLFG (TEC-A) or via consultation and general information (e.g., on their website and via staff members) by the SVLFG (TEC-A and ImplementIT) (Freund et al., 2020; Thielecke et al., 2020). The Consolidated Criteria for Reporting Qualitative Research (COREQ, Tong et al., 2007, see Supplement 1) and the Journal Article Reporting Standards for Qualitative Research (JARS-Qual, Levitt et al., 2018) have guided the reporting of methods and results in this study.

### 2.2 Involved researchers

The study design and interview guide were developed by psychologists with a master degree and some experience in interviewing (LB, JF, JT) under supervision of a clinical expert and qualitative research specialist (IT). Two students with a bachelor degree in psychology (JB, MM) conducted the telephone interviews and transcribed them. Both interviewers conducted a role-play test-interview and received feedback on their first participant-interview by JT.

The analysis and interpretation of data was conducted with a core team consisting of two psychology students (KB, VO) as part of their theses and the study team members of the ‘With us in balance’ project. The study team members had an education as psychologists or public health scientist and were employed in the project for multiple years. Study team members (mostly female) did not have vast experience of living/working in a farming context but were sympathetic and interested to it. Farming related knowledge was gained throughout the project by close collaboration with the SVLFG (e.g. shadowing counsellors during their work on farms as part of the project, regular project meetings with updates on farming specific news), however, it cannot be guaranteed that the farming context was always represented accurately.

The research team had no prior relation to the interviewees. The interviewees had contact with other psychologists (i.e. their coaches) before in a similar situation to the interviews when conducting the telephone coaching and might therefore be used to talk about mental health and the coaching to persons without framing background.

### 2.3 Intervention

All study participants took part in a personalized telephone coaching by IVPNetworks, which was financed by their insurance (SVLFG). The study enrollment process differed for TEC-A and ImplementIT participants, while coaching conduct was the same. Participants were registered on the IVPNetworks platform (IVPnet) by the university study team after submitting informed consent and being randomized to the intervention group (TEC-A) or by SVLFG call center agents after consultation about different services (ImplementIT). The case managers at IVP networks assigned the coach who then contacted participants.

A coaching volume of 850 min over six months was available for each participant with the possibility of additional 150 min over three months if approved by the insurance company. As part of the personalization of the coaching, session length and frequency of the coaching as well as topics were oriented on the individual participants needs. If indicated and available, on-site coaching or other support-services (e.g., socioeconomic counseling) could be arranged or recommended.

Coaches were psychologists with master's degree and training in diverse psychological methods (e.g., systemic, cognitive behavioral, hypnotherapeutic). No fixed manual was applied for the coaching. According to the coaches, typical elements of their coaching were psychoeducation, conjoint goal setting, and a three-phase model (‘introduction and alliance building,’ ‘working,’ and ‘stabilizing’ phase). Licensed psychotherapists were available for supervision. Intervention details can be found elsewhere (Freund et al., 2020; Thielecke et al., 2022, Thielecke et al., 2020).

### 2.4 Participants and recruitment

Interviews were conducted with a self-selected convenient sample recruited form the project ‘With us in balance’ not aiming for representativeness. Based on inclusion criteria for the studies (TEC-A, ImplementIT), all interviewees were 18 years or older, an agricultural entrepreneur (i.e., the official farm owner) or collaborating family member or pensioner and insured at SVLFG with access to internet and telephone. Interviewees recruited within TEC-A were required to have at least subclinical depression (PHQ ≥ 5).^7^ Participants could not currently receive psychotherapy and had to be able to distance from suicidal ideation. All participants provided extra written informed consent for the interviews, data-matching, and audio-recording.

Coaching participants (40/160, 25 %) from TEC-A were invited by mail in August and September 2019 who had stated initial interest in participating in the interview, had not withdrawn from the study in the meantime, and had completed coaching or would do so within a month. Out of 20 responders (50 %), 17 consented to the interviews, scheduled an appointment, and took part (42 %).

At the same time, coaches were asked to inform the 26 current participants of ImplementIT about the interviews and refer interested participants to the study team. Out of 12 responders (46 %), three consented to the interviews, scheduled an appointment, and took part (25 %). No incentives were offered as part of the interviews, but RCT participants could receive up to €45 for completing the RCT assessments (Thielecke et al., 2020). A sample of N = 20 was aimed for based on general recommendation (Francis et al., 2010; Hennink et al., 2017) and experience in the field with the potential for further recruitment if deemed necessary (e.g. because of low saturation). Descriptives of interviewees are presented alongside the RCT sample characteristics in order to allow for comparison between the study samples.

### 2.5 Development of interview guide

Questions for acceptance were based on the Unified Theory of Acceptance and Use of Technology (UTAUT^8^) (Venkatesh et al., 2003) which promoted the intention-to-use and actual use of technology. Questions for satisfaction were based on the eight characteristics of health care service described in Evaluation Model (Blum, 1998; Ware et al., 1983) in order to encompass participants' experience with a broad range of aspects. These characteristics comprise and specify the quality dimensions of structure, process, and outcome as suggested in Donadebian's classic Quality of Care Model (Donadebian, 2005). The Discrepancy Theory (Fox and Storms, 1981) informed the question on expectation fulfillment with the coaching, coach, and organization as an indicator for satisfaction (numerical rating form 0 % to 100 %). Table 1 presents the operationalization of all theoretic components and their adaptation to the telephone context in the interview guide. Interviews began with a brief outline of the interview procedure and the goal of the interview was described as the interest to learn more over the perception of the telephone coaching in order to improve the preventive offers of the SVLFG without naming the specific constructs of interest.

**Table 1:** Examples from the interview guide representing the theoretical components of acceptance and satisfaction with their adapted definitions used in the study.

Component	Definition	Example question
*Theory of acceptance and use of technology (acceptance)*		
Performance expectancy	Participant's beliefs on how the personalized telephone coaching is favorable for his/her professional or private situation	*When you think back to the beginning of the coaching: What expectations did you have regarding what should change through the coaching for you?*
Effort expectancy	Participant's expectations about the associated effort of the coaching	*How much time and work did you imagine you would have to put into the coaching?*
Social influence	Degree to which the social environment knows about the coaching and supports participation	*Which role did the support from friends and family play in your participation in the personalized coaching?*
Facilitating conditions	Participant's beliefs about what organizational and technical characteristics could support coaching participation	*What are the advantages [disadvantages] of a telephone coaching compared to a traditional on-site preventive service?*
Behavioral intention	The intention to use the personalized telephone coaching (again)	*How willing would you be to participate in another coaching?*

*Evaluation model (satisfaction)*		
Technical care quality	Competence of coach or his/her quality of care	*To what extent did you feel understood by your coach in terms of professional e.g., farming-related topics?*
Psychosocial care quality	Non-clinical aspect of coach-participant interaction (e.g., friendliness, empathy)	*How would you describe your relationship with your coach?*
Accessibility	Organizational conditions that support coaching participation	*Regarding the organization, which aspects were you especially [less] content with?*
Spatial and technical equipment	Spatial or technical difficulties during the coaching	*Which technical difficulties occurred during the coaching?*
Treatment outcome	Degree to which the coaching improved problematic situation or psychological burden	*What tangible changes came about in your everyday life through the coaching?*
Continuity of care	Continuity of coach	*Were you attended by the same coach the whole time?*
Financing	Financing of the coaching	*Would you have participated in the personalized telephone coaching if it was associated with costs for you?*
Availability	Availability of the personalized telephone coaching in care	*Would you also have chosen the telephone coaching if you had a comparable service available on-site?*

*Discrepancy model (satisfaction)*		
Expectation	Expectation regarding the coaching, the organization and the possible outcome	*When you think back to the beginning of the coaching: What did you expect from your coach?*
Expectation fulfillment	Discrepancy between expectations at the beginning of the coaching and real life conditions	*To what extent were your expectations fulfilled? Please name a percentage between 0 and 100* *%.*

The semi-structured interview guide contained 25 questions derived from the theoretical components (see Table 1) and organized in four blocks in order to give participants an easy to understand outline: ‘coaching process and result’, ‘coach’, ‘organization’ and ‘telephone delivery’. The first three blocks started with a question on the expectations participants had before the coaching began (e.g., in the block coach: “*When you think back to the beginning of the coaching: What expectations did you have regarding your coach?*”), which was followed by questions assessing details of the coaching process with regard to the theoretic components of acceptance and satisfaction (e.g. for the psychosocial care quality: “*Please think back to the coaching as a whole: How would you describe the relation with your coach?*”) and finally a rating question on how much of the beforementioned expectations were fulfilled (e.g., for the discrepancy theory with regard to the coach: “*To what extent were your expectations [regarding your coach] fulfilled? Please name a percentage between 0 and 100%.*”). The last block, focusing on the delivery mode focused on three questions asking about advantages and disadvantages of the telephone delivery (e.g., “*What [advantages/disadvantages] do you see with telephone coaching compared to a traditional on-site prevention service?*”) and the intention to use (e.g., “*How willing would you be to participate in another coaching?*”). Additionally, 21 memo questions (e.g., “*How has your relationship with your coach changed over the course of the coaching?*”) and inquiry (e.g. “*What else comes to mind about your coach?*”) questions were given to ensure complete and detailed answers.

### 2.6 Data collection

#### 2.6.1 Qualitative data collection

Interviews were conducted mostly after the end of the coaching via telephone between August and October 2019. Average interview duration was M = 44 min (SD = 17 min). Interviews were audio-recorded using PhonerLite (Sommerfeld, 2019) and audacity (Audacity Team, 2021) and transcribed verbatim according to a transcription protocol. Names were anonymized during transcription.

#### 2.6.2 Quantitative data collection

Sociodemographic data were assessed online for interviewees of ImplementIT or were extracted from TEC-A baseline assessment. For later comparison and a triangulation approach (Heale and Forbes, 2013), data on depressive symptom severity (QIDS-SR16,^9^ Quick Inventory of Depressive Symptomology, Rush et al., 2003, range 0–27), intervention satisfaction (CSQ-I,^10^ adapted Client Satisfaction Questionnaire for Internet-based Interventions, Boß et al., 2016, range 8–32), and working alliance (WAI-SR,^11^ Working Alliance Inventory - Short Revised, Munder et al., 2009, range 0–5) were derived for RCT data as well.

After the qualitative analyses of the interview material, interviewees were invited to provide feedback on the identified themes in August to September 2021 via an online validation questionnaire indicating their agreement with ‘yes’ or ‘no’.

### 2.7 Data analyses

A qualitative content analysis was conducted to summarize and structure the material following the general approach by Mayring (2015) and adapted to fit the research question of identifying aspects potentially influencing satisfaction and acceptance.

(1) Text passages relevant to the theoretic components of acceptance and satisfaction were identified and paraphrased in a deductive way for 10 % (n = 2), 20 % (n = 4), 50 % (n = 10) of the material, consecutive until no new topics arose for the last interviews (code saturation, Saunders et al., 2018). Basis for this was the full-length interview. Context entity was the full statement with the smallest coding entity being one sentence. Discussing paraphrases (KB, VO, JT) let to the definition of broad dimensions, sometimes combining theoretical components which were shared by both acceptance and satisfaction.
(2) From the paraphrases, a list of possible codes was stepwise developed (by generalizing the excerpts) and inductively organized in (sub)-themes under the dimensions until encompassing 50 % (n = 10) of the total material. Codes, definitions, and example statements were discussed in regular consensus meetings (KB, VO, JT).
(3) After coding 50 % of the material, feedback on coded passages was given (JT) and (sub-)themes were rearranged and sharpened in definitions by JT and CB. In this process, CB and JT decided to no further include the theoretical components ‘ continuity of care’, ‘financing’ and ‘availability’ in the analysis because thus far participants answered related questions either too short or only described the status quo without evaluating it. Therefore, no insight could be gained on how these components influenced satisfaction. A preliminary code system was formed addressing themes which were allocated to supporting (SF) and hindering factors (HF) over the five cross-theory dimensions ‘Coaching Result’, ‘Coach’, ‘Organization’, ‘Participant Characteristics’ and ‘Telephone as Communication Medium’. While most dimensions entailed aspects related to both constructs, the dimension ‘Coach’ related solely to the theoretic components ‘Technical’ and ‘ Psychosocial care quality’ of the evaluation model of satisfaction and the dimension ‘ Participant Characteristics’ represented exclusively the aspects ‘Behavioral intention’ and ‘Social support’ of the UTAUT model for acceptance.
(4) The preliminary code system was again tested on 50 % (n = 10) of the interviews (KB, VO), coded passages feedbacked (JT) and the code system reworked after consensus meetings (KB, VO, JT).
(5) A first coding of the full 20 interviews by both coders (KB, VO) revealed the necessity to redefine definitions of three sub-themes and add one emerging additional theme in order to clearly distinguish themes and encompass the full material.
(6) The second, final coding of 100 % material (N = 20) was conducted by two independent raters (KB, VO). High agreement (Landis and Koch, 1977) between the two raters was reached indicated by Cohen's kappa k = 0.80.
(7) Two themes describing hindering aspects were named solely ones (4 % of themes). This diminishes meaning saturation since themes derived from only one participant might encompass it less comprehensive (Fusch and Ness, 2015; Hennink et al., 2017). All other themes (96 %) were named at least two times.
(8) For a higher fit of mapping codes to the theoretical background, the five cross-theory dimensions were critically revised without changing definitions or coded passages (CB, IT, JT). For every dimension supporting and hindering factors were identified. Expectations were identified for dimensions ‘Coaching Result’ and ‘Coach’.
(9) Participants were invited to provide feedback on the identified themes in August to September 2021 via an online questionnaire.

Supplement 2 illustrates the whole study process including data collection and analysis. Analyses were conducted using MAXQDA 2020 (Sozialforschung GmbH, 2021) for qualitative and R 4.1.0 (R Core Team, 2020) and Excel (Microsoft Corporation, 2018) for quantitative data.

## 3 Results

### 3.1 Participant characteristics and intervention use

Half of the interviewees were male (n = 10, 50 %), most lived in partnership (n = 18, 90 %), had middle education (n = 14, 70 %), and were, on average, 55 years old (SD = 8.62). Interviewees recruited from TEC-A reported high satisfaction with the intervention (M = 30.06, SD = 3.75) and a good working alliance with their coach (M = 4.30, SD = 0.48) six months after study begin. Interview participants showed comparable characteristics to RCT coaching participants in demographics (Table 2) and intervention use (Table 3) but cannot be understood as a representative sample. Time between end of coaching and interview varied between two weeks and six months with two interviewees (10 %) still in the prolonged coaching at time of the interview.

**Table 2:** Interview participants' characteristics compared to not interviewed coaching participants in the RCT TEC-A.

Variable	Interview participants (n = 20)			Not interviewed coaching participants in TEC-A (n = 143)		
	M	SD	Range	M	SD	Range
Age	55.00	8.62	31–74	52.87	9.30	30–78
Depressive symptom severity (QIDS-SR16)^a^						
At baseline	9.06	3.68	4–16	9.90	4.19	0–24
After 6 months	5.50	3.81	1–14	6.52	3.91	0–20


Variable	Interview participants (n = 20)		Not interviewed coaching participants in TEC-A (n = 143)	
	n	%	n	%
Sex				
Male	10	50	75	52
Female	10	50	68	48
Birthplace				
Germany	20	100	141	99
Other	0	0	2	1
Relationship status				
No partnership	2	10	10	7
With partnership	18	90	133	93
Children^a^				
Children	15	88	128	90
No children	2	12	15	10
Education level^b^				
Low	0	0	14	10
Middle	14	70	91	64
High	6	30	38	26
Profession				
Entrepreneur	13	65	84	59
Entrepreneurs spouse	4	20	31	22
Pensioner	2	10	14	9
Family member	1	5	13	9
Incapacitated for work	0	0	1	1
Main branch of produce^a^				
Horticulture	4	21.05	8	6.11
Animal husbandry	3	15.79	30	22.9
Milk production	3	15.79	31	23.66
Wine production	3	15.79	16	12.21
Fields/greenland	2	10.53	26	19.85
Vegetables	2	10.53	7	5.34
Other^c^	2	10.53	13	10.74
Business size a				
1–4 persons	10	52.63	70	53.44
5–9 persons	6	31.58	38	29.01
10–24 persons	3	15.79	20	15.27
25–49 persons	0	0	1	0.76
50–99 persons	0	0	2	1.53
100 or more persons	0	0	0	0
Experience with psychotherapy^a^				
No experience with psychotherapy	14	82	115	80
Experience with psychotherapy	3	17	28	20

Note: Abbreviations: T0: baseline assessment study begin; T1: post assessment 6 months after randomization; QIDS-SR16: Quick Inventory of Depressive Symptomology (range 0–27).

a Data only available for participants in the associated randomized controlled trial (RCT, study acronym: TEC-A – telephone coaching for agriculturists; T0: n_interviewed RCT participants_ = 17, n_not interviewed RCT participants_ = 143; T1: n_interviewed RCT participants_ = 16, n_not interviewed RCT participants_ = 121).

b Education levels defined as: low = under 10 years of formal education, middle = at least 10 years of formal education, high = at least bachelor degree or equivalent.

c Other category summarizes all categories with <5 namings.

**Table 3:** Intervention related characteristics for interviewees and not interviewed coaching participants in RCT TEC-A six month after study begin.

Variable	Interview participants (n = 20)			Not interviewed coaching participants in TEC-A (n = 143)		
	M	SD	Range	M	SD	Range
Days from enrollment to first contact by coach^a^	2.41	2.81	0–11	3.15	3.41	0–13
Number of sessions	15.40	4.59	6–24	13.18	6.10	1–32
Coaching volume in minutes	732.30	239.33	265–1057	643.07	332.26	50–1598
Duration in weeks	25.74	6.37	10.29–44.43	24.39	8.09	1.43–38.86
Participant satisfaction (CSQ-I)^a^	30.06	3.75	18–32	27.92	5.75	9–32
Working alliance (WAI-SR) total^a^	4.30	0.48	3.5–5.0	4.02	0.68	1.42–5.0
WAI-SR bond	4.48	0.42	3.5–5.0	4.25	0.70	1.5–5.0
WAI-SR task	4.16	0.52	3.0–5.0	3.77	0.78	1.0–5.0
WAI-SR goal	4.27	0.69	3.0–5.0	4.05	0.75	1.0–5.0
Days between coaching end and interview^b^	41.44	46.68	5–191			


Variable	Interview participants (n = 20)		Not interviewed coaching participants in TEC-A (n = 143)	
	n	%	n	%
Prolonging of coaching				
No	18	90	115	81
Yes	2	10	27	19
Documented discharge reason				
Improved/stabilized quality of life	14	70	84	59
Stabilized and further support recommended/arranged	6	30	42	30
Intervention dropout				
Withdrew consent for intervention	0	0	7	5
Lack of compliance	0	0	5	3
Intervention not fitting	0	0	3	2
Other	0	0	1	1

Note: Session information only reported for participants with at least one coaching session (n_interviewed partcipants TEC-A and ImpleMentIT_ = 20, n_not interviewed RCT participants_ = 142). Abbreviations: CSQ-I: Client Satisfaction Questionnaire for Internet-based Interventions (range 8–32); WAI-SR: Working Alliance Inventory - Short Revised (range 0–5).

a Data only available for participants in the associated randomized controlled trial (RCT), study acronym: TEC-A – telephone coaching for agriculturists (n_interviewed RCT participants_ = 17, n_not interviewed RCT participants_ = 119).

b n = 18 since two participants were still in coaching, final intervention data reported in other variables.

### 3.2 Aspects influencing acceptance and satisfaction

In total, eight expectations (E), 32 SF, and 14 HF that potentially influenced acceptance and satisfaction were identified and organized under five cross-theory dimensions. The dimension ‘Coaching result’ entailed a description of expected changes or improvements sought from the coaching (E = 4), supporting factors (SF = 9), which were sorted into two broader themes and hindering factors (HF = 3). SFs described actual changes initiated by coaching (which included sub-themes like ‘more acceptance and calmness’ or ‘improved self-care’) and effective factors of the coaching. Aspects named as hindering to achieve the desired changes included a perceived continued need for support and need for more exercises and tips (HF = 3). In the dimension ‘Coach’, expectations (E = 4) were mostly concerned with the coach's professional competence, while the SFs entailed the broader themes coaching relationship and coach characteristics as well as their competence (SF = 9). The sole HF related to the dimension ‘Coach’ was ‘low of agricultural knowledge’. Under the dimension ‘Organization’ (SF = 5, HF = 2) the flexibility, quick availability, and regularity of appointments as well as the individual time frame and invitation letter were identified as SFs. The coaching duration perceived as too short or appointments within core working hours were identified as HFs. The dimension ‘Participant Characteristics’ included the broader themes of attitudes toward telephone coaching (including the sub-theme ‘preference for telephone coaching’) and social support (SF = 5), while HFs encompassed lack of time, preference for other services and negative social reactions (HF = 3). Lastly, the dimension ‘Telephone as Communication Medium’ covered the four SFs of perceived low effort, a sense of anonymity, the value of a familiar environment during the coaching and the lack of distraction with telephone delivery. Hindering factors concerning the telephone conduct (HF = 5) included the lack of visual cues, technical difficulties, perceived restriction in the coaching conduct and coaching relationship and a lack of distance to the problems.

Table 4 gives a summary of all identified themes with definitions and quotas in the form of the coding system used for the analyses. An overview on all themes, cross-theory dimensions and their relation to the theoretical frameworks can be found in Supplement 3. The most often reported themes for expectations, supporting and hindering factors for each cross-theory dimension are described below and illustrated with a quote.

**Table 4:** Overview of all identified themes, frequencies, definition, and example statement as used in the coding system.

Theme	n	%	k	Definition	Supporting quote
*Coaching result*					
Expectations (Q = 4, k = 27)					
Improvement of psychological symptoms	9	45	11	Participants expected an improvement of clearly named psychological symptoms (e.g., depression, anxiety, psychosomatic or neurological complaints, restlessness, stress) through the coaching.	“*The second reason was that I have been stuck in a burnout for years or, how should I say it, that I can't get back on my feet.*” (p7)
Desire for change	9	45	11	Participants express a nonspecific desire for change, overall improved well-being or (re-)gain of balance.	“*But regaining balance and that was, at the start, kind of my motivation or demand and hope that I had in it.*” (p10)
Support in difficult phases	2	10	3	Participants expected that by participating in the coaching, they would receive support in difficult phases in order to prevent even worse consequences.	“*Well, what was important to me is that I am somehow guided through this extremely exhausting phase because in spring it's [harvest] season and I was thinking ‘I can't do all this!’”* (p1)
Improved self-management	2	10	2	Participants expected to achieve better self-management or self-control through the coaching.	“*My goal or my wish was: (…) I want to get broad shoulders. That I don't always cave in and start crying, sometimes it worked but sometimes it's still like that.*” (p1)
Supporting factors (Q = 9, k = 156)					
Effective factors for coaching					
Concrete coaching methods	18	90	31	Participants name or describe concrete coaching methods such as breathing exercises, cognitive restructuring, take-home exercises or journaling as helpful for achieving results.	“*She [the coach] also breathed with me. When I started to fall back into old behavior patterns, during the conversation as well. That honestly helped me the most.*” (p6)
Opportunity for conversation	16	80	23	Participants rate the opportunity to have a conversation in itself or talking about problems/topics as a conducive factor to achieving the coaching result.	“*Well, definitely the conversation. That contributed to it, simply because it gave me suggestions (…) and a positive feeling.*” (p2)
Setting realistic goals and doing exercises	6	30	6	Participants name realistic goal setting as well as exercises during the coaching as helpful.	“*But it wasn't like there were any ideas, let's say (…) ‘This can't go on like this, you have to take a 4-week vacation. Do that immediately.’ I wouldn't have been able to execute (…). Everything we talked about was doable.*” (p8)
Defining and reflecting on the problem	5	25	8	Participants name describing the problem, gaining awareness for the problem, and consciously dealing with one's own situation as helpful for achieving the coaching result.	“*What always helped me a lot was that you could describe the problem precisely and find approaches for how to escape the vicious circle and that helped me a lot.*” (p10)
Changes initiated by coaching					
More acceptance and calmness	14	70	24	Participants report having developed more acceptance for certain aspects in life and more calmness or self-control as a result of the coaching.	“*Well additionally, the main change was actually making me more relaxed, gaining more distance between me and my company, that I can evaluate things better*” (p4)
Improved self-care	11	55	26	Participants report an improved self-management as a result of the coaching, which includes improved self-care as well as increased self-confidence.	“*Just that I'm more calm, more selfish, that I can lead conversations with my coworkers differently, that I am more understanding in certain situations with my coworkers.*” (p4)
Reevaluation of situations	10	50	17	Participants report actually evaluating situations differently as a result of the coaching instead of merely being shown a different view by the coach.	“*In my daily life - In principle nothing changed. But my attitude toward things has changed.*” (p6)
Improved overall and psychological well-being	9	45	15	Participants report having improved their overall or psychological well-being through the coaching (specifically mental health problems such as sleeplessness, stress, or depressive symptoms and nonspecific improvements of well-being).	“*I would say that you gain a certain distance to your job or company, that you also have periods of rest, your own breaks, that you sleep through the night again and your thoughts don't continue rattling.*” (p10)
Improved and more open communication	4	20	6	Participants report having improved communication with their social environment and talking more openly about private and health-related topics as a result of the coaching.	“*As I said, overall, that I became more calm, that I can talk more easily about the illness.*” (p3)
Hindering factors (Q = 3, k = 10)					
Perceived continued need for support	5	25	5	Participants still see a need for support after the coaching and wish for specialized counseling centers, aftercare, and outpatient or inpatient psychotherapy or group services.	“*It could get more concrete, that you would, for example, get recommended two or three points of contact that have already proven themselves in the field. (…)*” (p17)
Expected results not achieved	2	10	2	As a negative aspect, participants name not achieving the expected coaching results.	“*For me, it was primarily about finding out the reasons for my depressive phase. And for that, I believe, the amount of time was too short.*” (p5)
Lack of exercises and tips	1	5	3	Participants describe a lack of exercises and tips for implementation in daily life.	“*Yes I actually wished I would have gotten a few practical little instructions. Along the lines of, (…) Let's say, when I'm totally stressed, take 5 deep breaths or something like that, these practical instructions, [were missing]*” (p8)

*Coach*					
Expectations (Q = 4, k = 32)					
Professional counseling	9	45	12	Participants expected that the coach would support them through professional counseling and assessment of their situation and that they would give them practical tips, advice, and exercise suggestions.	“*(…) I have a case of mental illness in my family. My brother suffers from severe depression and that burdens me greatly and I hoped to get advice from the coaching on how I should act in front of my brother.*” (p9)
Solution-oriented problem discussion	7	35	8	Participants expected from the coach that they would discuss different topics/problems with them (in a solution-oriented manner).	“*(…) That I could talk about it, that I could get new ideas and support so that I could categorize it.*” (p10)
Empathetic listening	5	25	7	Participants expected from the coach that they would actively listen and respond to the participants in an empathetic way.	“*The expectation that he would take what I say seriously, that we can talk on eye level. And (…) that he would respond to what I'm telling him.*” (p2)
Professional competence	5	25	5	Participants expected that coach would be competent in their sphere.	“*That he [the coach] understands his job. (…) I actually assumed that someone would be chosen who knows exactly what is expected from him.*” (p4)
Supporting factors (Q = 9, k = 115)					
Competence of coach					
Empathy	11	55	24	Participants perceive the coach's empathy which includes aspects that take place on an emotional level (e.g., understanding the other person and being genuinely interested in the individual's situation) as positive.	“*I mean, I have a hands-on profession and I am also not the only, let's say patient, she had similar conversation before and after me, and that's unbelievable how someone can respond to a person in such a way and give them the feeling, you're not one of many, but I know exactly how you mean it and I'm going to help you solve this.*” (p4)
Conversation skills	10	50	22	Participants perceive the way the coach led conversations (i.e., asking targeted questions, activating and steering the conversation in a solution-oriented direction) as positive without describing concrete coaching methods.	“*I was not told, you have to do it a certain way, not that, but she did make sure that I develop [my own thoughts] and then she dug deeper in the right places.*” (p1)
Encouragement of participants	5	25	11	Participants perceive the coach's positive reinforcement in the form of praise, encouragement, and validation of one's own statements as positive.	“*Just through the conversation and that she encouraged what I said myself (…).*” (p1)
Agricultural knowledge	5	25	7	Participants perceive the coach's agricultural knowledge as agreeable.	“*Well, it wasn't overly important, but it was quite nice that there was certain background knowledge and the coach was also interested in things I told him about agriculture.*” (p2)
Coaching relationship					
Trusting relationship	16	80	23	Participants describe their relationship with the coach as trusting/secure or friendly/comradely.	“*The relationship was relaxed, trusting.*” (p2)
Demographic commonalities	5	25	6	As a conducive factor in relation to the coach, commonalities in demographic data (e.g., sex, ethnicity, age, etc.) are named.	“*I also thought about whether I would be able to talk to my husband as well as with her, I probably would, but it was actually nice being able to have a woman-to-woman talk.*” (p4)
Professional relationship	4	20	4	The relationship with the coach is described as professional/respectful.	“*Well, it was a good relationship but not in the sense that I was dependent on her. I did think, well you have to make sure that you remain in control of yourself and don't make her responsible for your happiness. There was a clear separation between what we both did professionally and privately.*” (p4)
Picture of coach	3	15	3	Participants rate the coach's picture that was previously sent to them per e-mail as positive.	“*(…) Maybe I still belong to the older [generation] who sometimes still want to know who's on the other side of the phone if you're calling almost every week (inaudible). Therefore I thought the picture was quite nice (…)*” (p10)
Coach characteristic					
Independence of coach	10	14	15	Participants rate the coach's independence/objectivity as positive.	“*And it's always better to talk to someone independent.*” (p3)
Hindering factors (Q = 1, k = 9)					
Low agricultural knowledge	4	20	9	Participants rate the lack of knowledge in the agricultural sphere on the part of the coach as negative.	“*It was maybe just the technical knowledge. He [the coach] took a lot of what I gave him from the professional sphere. There was maybe this 1 % left, because he didn't operate that job-specific.*” (p2)

*Organization*					
Supporting factors (Q = 5, k = 85)					
Flexibility of appointment arrangement	19	95	35	Participants describe the compatibility with their professional and private duties, e.g., thanks to a flexible arrangement and postponement of appointments. This way the coaching can be successfully integrated in daily life.	“*It all went very well. Well, we arranged appointments and if you couldn't make them for once, then you quickly got in contact with each other. It all went very smoothly, well I was very satisfied*.” (p5)
Quick availability	13	65	23	Participants rate the quick availability and the short waiting times as positive.	“*In terms of processing speed, starting with my first call and then how quickly the whole thing was organized, I can only say that I was surprised how quick it went.*” (p6)
Regularity of appointments	10	50	17	Participants rate the reliable appointments and the regularity of appointments as positive.	“*Well, that we almost always talked on the same day and time, with a few exceptions. I could rely on that.*” (p3)
Personal invitation by mail	5	25	6	Participants rate the personal invitation to the coaching by postal letter from the social insurance company (at the right time) as positive.	“*So I was contacted by the agricultural social security that there is this study and that they offer me this and because I have just been under extreme stress at the moment because my husband passed away in October and I had to take care of the gardening myself, this came at the perfect time.*” (p1)
Individual time frame	4	20	4	Participants rate the individual time frame (e.g., orientation toward individual need and overall length) of the coaching as positive.	“*And that it went on for a longer period of time, not only three or four calls, but that it went on for a longer period of several months.*” (p2)
Hindering factors (Q = 2, k = 12)					
Coaching duration too short	5	25	10	Participants describe the overly short duration of coaching as negative.	“*It's just that this whole thing was a little too short because these things take time, now I can take care of it a little more (…)*” (p10)
Appointments within core working hours	1	5	2	Participants describe appointments within core working hours as an aggravating factor.	“*As I touched on just now, sometimes appointments after work would be more relaxed because I don't have anyone behind me wanting something from me.*” (p10)

*Participant characteristics*					
Supporting factors (Q = 5, k = 52)					
Social support					
Supportive and encouraging reaction from environment	16	80	19	Participants report positive or supportive and encouraging reactions from their environment.	“*Well my family had a positive view. When we talked about it, they definitely supported me and said, this is a good thing, you should continue if it makes you feel good.*” (p2)
Active integration of environment in the coaching	10	50	10	Participants report an active integration of their social environment in the coaching, or the coaching opened possibilities for conversations.	“*The support was just that I repeated what Ms. [anonymized] and I discussed during the coaching when I was my family.*” (p6)
Attitude toward telephone coaching					
Preference for telephone coaching instead of on-site service	11	55	11	Participants would have chosen the telephone coaching even if there had been a comparable service on site.	“*At the time, I would have always preferred the telephone coaching because of the time aspect. The other wouldn't have been viable at that point in time. And nowadays I would say the same.*” (p10)
Willingness to find time for coaching	5	25	8	Participants explain that a willingness to take time for the coaching is important.	“*This was possible because I said: ‘I need this time now, I have to take time off, I'm gone now’.*” (p4)
Willingness to engage in the coaching	3	15	4	Participants talk about how it is necessary to open up to a stranger and an unfamiliar situation and have to admit to your own problems.	“*But when I got accepted to this program of individual coaching sessions, in that moment, it was clear to me that I have to and want to open up to a complete stranger on the phone.*” (p6)
Hindering factors (Q = 3, k = 32)					
Social/professional involvement restricts change process	10	50	16	Participants describe being subject to their work life, their social environment, and the lack of possibilities as hindering to achieving results.	“*In the professional sphere, it's difficult because I'm trapped in my role as a wife and company manager and I can't leave just like that.*” (p9)
Preference for on-site services	7	35	13	Participants would have preferred a comparable on-site service over telephone coaching.	“*If the comparable on-site service had been a personal conversation, I would have definitely chosen that.*” (p7)
Negative reactions from one's social environment	3	15	3	Participants report negative reactions from their social environment in response to their participation in the coaching.	“*I didn't look at it in that way, but there were also my husband's preconceptions. (…) ‘That they [the insurance] will use it against you afterwards?’*” (p18)

*Telephone as communication medium*					
Supporting factors (Q = 5, k = 70)					
Low effort	17	85	42	The perceived advantage of telephone coaching was low time, organizational and financial effort (compared to on-site services).	“*You don't have to drive anywhere, even if you were at the shed earlier, you don't have to shower or change, but instead you can just sit in your office in work clothes and you can make the phone call. This isn't possible with a personal coaching.*” (p5)
Feeling of anonymity	10	50	10	Participants name the feeling of anonymity and low inhibition threshold as an advantage of telephone coaching.	“*Maybe there are people who are better at talking over the phone, who can open up more easily. Well for those people that would clearly be an advantage, right?*” (p7)
Familiar environment during coaching	9	45	11	Participants perceive the familiar environment during telephone coaching as positive.	“*There was the time aspect and actually the familiar environment which made me feel safe to some extent.*” (p11)
No distraction	5	25	7	Participants perceive the absence of distracting external factors (e.g., external appearances, stimuli in the treatment room) as conducive to focused conversation, concentration on the essentials, and unbiased participation in the conversation.	“*Well it was definitely good that it was neutral, because I think you subconsciously judge people by their appearance, and this way that [aspect] was eliminated and you could only hear your counterpart but you couldn't see them.*” (p4)
Hindering factors (Q = 5, k = 42)					
Lack of visual clues	12	60	20	Participants name the loss of visual clues (e.g., absence of eye contact and nonverbal communication) as a disadvantage of telephone coaching.	“*Well the disadvantages are clearly that you can't see your counterpart. I do find it different. For some people, the inhibition threshold to talk about something might be lower, but I would find it comfortable if you could sit across each other and talk properly.*” (p8)
Technical difficulties	7	35	7	Participants report technical difficulties during the coaching.	“*There were indeed problems with the connection being gone, sometime we have that.*” (p8)
Less trusting coaching-relationship	4	20	5	Participants mention the difficulty of opening up and building trust with the coach as a potential disadvantage of telephone coaching.	“*I was a little concerned whether you could establish a trusting relationship over the phone as opposed to actually sitting or standing across from each other, eye to eye so to speak, but it actually turned out to be unproblematic.*” (p17)
Restrictions in coaching conduct	4	20	6	Participants suspect that not all techniques and approaches that are applicable to telephone coaching are applicable on site.	“*That you can maybe not use certain techniques, I wouldn't know about that. (..) That there are certain techniques that you can't communicate over the phone.*” (p5)
Lack of distance from problems	3	15	4	Participants describe remaining in a familiar environment and the lack of distance to the problem during the coaching as hindering to the coaching process.	“*That it takes place in the usual space. That you're not seperated. (..). Well personally, I'm never seperated when I'm in a private, personal space, in my usual environment. But I can seperate myself within seconds when I'm in an unfamiliar atmosphere.*” (p7)

In supporting quotes, pauses and comments removed from quotes for better readability. (…) indicate left out parts without changing the content of the sentence. [..] indicate complements and explanations derived from the context of the quote. Abbreviations: n = number of participants naming the theme; % = percentage of participants referring to the N = 20 interviewed persons; k = number of coded elements in the interview data; Q = number of themes identified on the level of a dimensions.

#### 3.2.1 Coaching result

As expectations toward the coaching result, most often interviewees reported either *improvement of psychological symptoms* and/or a *general desire for change* (each n = 9, 45 %). An interview quote illustrating these themes is as follows:

‘*I think, I generally had the hope that I can find a way out of this dark thinking patterns. So, I did not really have precise expectations in that sense, but I really had this kind of basic need, that this darkness can become lighter.*’

(p11)

According to participants, *concrete coaching methods* such as breathing exercises and goal setting exercises (n = 18, 90 %) were most helpful to reach their goals.

‘*For example, I am looking at a table right now: (…) what I want to do differently, then when I start with it and how I reward myself when I completed it.*’

(p4)

Less HFs were identified that could have influenced satisfaction with the coaching result. A quarter of interviewees (n = 5, 25 %) described *perceived continued need for support* ranging from wanting information about further help services to starting face-to-face therapy and hence indicated that their desired goal was not completely met:

‘*That you could at least have a therapy place once a month or here [on-site].*’

(p3)

#### 3.2.2 Coach

With regard to their coach, participants most often expected a person that can deliver *professional counseling* and give advice in their situation (n = 9, 45 %).

‘*That there is somebody, who can give me hints or advice, how I can get out of this situation in which I was in at that point (…).*’

(p11)

In terms of SFs, most of the participants (n = 16, 80 %) described to have experienced a very *trusting relationship* with their coach:

‘*That developed over time and then it was, well, trusting and a very good relationship, but also respectful and friendly.*’

(p1)

Only one HF, namely *low agricultural knowledge* of the coach, was named by four participants (20 %).

‘*When we talked about my role in the family and on the farm (…) it would have been helpful if she had a bit more insight.*’

(p9)

#### 3.2.3 Organization

The most named (supporting) theme of all (n = 19, 95 %) was flexibility of appointment arrangement which facilitated participation. Interviewees described an easy integration of scheduling into their everyday life:

‘*If I was really busy on the field for once, one postponed the appointment. That worked even on relative short notice.*’

(p5)

A quarter of participants (n = 5) perceived the *coaching duration as too short* to achieve their goal and that possibly diminishing satisfaction:

‘*Because with half a year, you are not simply out of the problem like that.*’

(p15)

#### 3.2.4 Participant characteristics

The most often named SF on participant level was supportive and encouraging reaction from environment (n = 16, 80 %):

‘*My wife thought it was really great that I was doing something like that.*’

(p5)

Half of the participants described their high *social and professional involvement* as restricting for the change process initiated by the coaching and as hindering for achieving their goals (n = 10, 50 %):

‘*That you're so tied up in the job that there's a lack of opportunity.*’

(p7)

#### 3.2.5 Telephone as the communication medium

Most controversial topic was the telephone as the communication medium yielding most HFs (n = 5) but likewise four SFs. Most named supporting factor was the perceived low effort through telephone delivery (n = 17, 85 %):

‘*That was crucial for me – that it was over the phone. I would not have gotten into a car and driven somewhere for a quarter of an hour. (…)*’

(p1)

Over half the participants reported the *lack of visual cues* via telephone conversation as the most common hindering factor for the coaching conduct (n = 12, 60 %):

‘*Except that you can't see whether the coach is listening or sometimes a bit of facial expression or certain feedback (…). That is missing a bit.*’

(p20)

### 3.3 Degree of satisfaction

Satisfaction with the intervention was indicated by the numerical ratings on expectation fulfillment. The above reported expectations toward the coaching result (n = 20, M = 85 %, SD = 16 %) and the coach (n = 20, M = 96 %, SD = 7 %) were met to a high degree. Even though no expectation but actual experiences were named with regard to the organization, fulfillment were rated high as well (n = 18, M = 99 %, SD = 3 %).

### 3.4 Results of the validation questionnaire

All interviewees were invited and 17/20 (85 %) responded. In the validation questionnaire, on average, 66 % (range: 0 %–100 %) of participants agreed to the identified themes: Agreement rates were higher for expectations (M = 87 %, range 56 %–100 %) and SFs (M = 81 %, range: 44 %–100 %) than for HFs (M = 20 %, range 0 %–50 %). Supplement 3 provides a comparison of qualitative coding results and validation.

## 4 Discussion

In this theory-based interview study, farmers participating in a personalized telephone coaching to prevent depression were asked for their experiences with the intervention in order to conclude factors that might influence their acceptance and satisfaction.

### 4.1 Main findings

The influencing factors identified were organized under the dimensions ‘Coaching Result’, ‘Coach’, ‘Organization’, ‘Participant Characteristics’, and ‘Telephone as Communication Medium’. More than half of interviewees stated to have preferred the telephone offer over a comparable on-site intervention from the beginning, and all participants used the telephone coaching for at least 10 weeks, which indicated acceptance for the intervention. The interviewees reported an almost complete fulfillment of their expectations for the coaching, which indicated their satisfaction with the intervention. Correspondingly, twice more supporting than hindering factors to satisfaction and acceptance were identified, indicating positive experiences by all interviewees.

With regard to the theoretic components, codings for the dimension ‘Coaching Result’ (e.g., changes initiated by the coaching), the ‘Organization’ (e.g., flexibility in conduct), and the ‘Telephone as Communication Medium’ medium revealed SFs and HFs for both acceptance and satisfaction. Participants' experiences with respect to the dimension ‘Coach’ and especially the good coach-participant relationship primarily supported satisfaction with the intervention, while reported ‘Participant Characteristics’ (having a positive attitude and social support) mainly facilitated acceptance.

Across all dimensions, multiple themes indicated a perceived personalization as another, overarching supporting factor for the acceptance and the satisfaction with the telephone coaching, which can be summarized with regard to the following aspects:

(1) Individual time composition: Alongside the reduced effort by eliminating traveling times, the flexible arrangement of appointments was highlighted that allows for an easy integration into everyday life.
(2) Appreciation for occupational background: The study invitation, addressing common stressors in farming occupations, was perceived as very personal by interviewees. Coaches' agricultural knowledge, if present, was perceived as supporting.
(3) Adaptation of coaching methods: The reported realistic goal setting and exercises allowing for constrains by interviewees' work life reflect a personalized procedure in this intervention.

The remote delivery format via telephone was viewed divisive by interviewees. Almost all participants highlighted the perceived low effort of the telephone coaching (e.g., no need to change clothes or travel) and half of the interviewees valued the feeling of (visual) anonymity and staying in a familiar environment during the telephone coaching. On the other hand, telephone-related disadvantages (e.g., lack of visual clues, technical problems) were the most reported hindering aspects and deemed to diminish the coaching conduct or effectiveness in participants' views.

The results of the validation questionnaire supported the interpretation that the HFs named only by a few interviewees (e.g., coaching being not practical enough or too short) were indeed rare as average agreement rates for these were low.

### 4.2 Comparison with literature

The described participants' experiences with personalized telephone coaching are very similar to earlier studies reporting experiences by MDD patients with more standardized CBT over telephone. These reflect the advantages of the telephone use (e.g., convenience, anonymity) as well as potential problems of it (e.g., technical problem, lack of visual feedback) (Bee et al., 2010; Haller et al., 2019). The analysis of our interviews with farmers added to the research field the aspects of the flexibility and adaptation to occupational background as facilitating for intervention use. Despite sporicidal skepticism, the good therapeutic relationship reported in our and earlier studies (Bee et al., 2010; Haller et al., 2019) is in line with studies reporting no difference in therapeutic relationship in telephone- or face-to-face therapy (Irvine et al., 2020; Stiles-Shields et al., 2014).

The findings in this qualitative study are coherent with the quantitative results observed in the associated RCT (Thielecke et al., 2022) that suggested good acceptance (e.g., only 11 % intervention dropout) and high satisfaction with the intervention (CSQ-I: M = 28.17, SD = 5.58) in farmers. Aligned with the experienced changes initiated by the coaching (e.g., more acceptance, improved self-care) reported by the interviewees, the RCT showed a reduction in depressive symptom severity and further mental health outcomes including stress and anxiety. Similarly, the stated supporting aspects of a good coaching relationship and a realistic goal setting in the interviews corresponded to the observed ratings on the subscales of the working alliance questionnaire in the RCT.

Both the UTAUT model and the theories for patient satisfaction could be applied to our remote health care setting with slight adaptations. Some aspects seem to be especially worth looking at with regard to our farming sample:

In terms of acceptance, perceived low effort and quick availability when compared to on-site psychotherapy (Bundespsychotherapeutenkammer, 2019) were often named by interviewees, suggesting those factors might support the uptake of mental health care. Researchers on telehealth interventions have argued that remotely delivered health service might offer low-threshold access to (mental) health care (Ebert et al., 2018; Snoswell et al., 2021) and improves health care in underserved areas (Myers, 2019; Salize et al., 2007), which is supported for farming communities by our interviewees.

For the experienced satisfaction with the intervention, the aforementioned personalization of the intervention seems to be another promoting factor for our farming sample.

Given time restrictions by often long and unpredictable working hours in this occupational group (Bowyer et al., 2023; Braun et al., 2022; Daghagh Yazd et al., 2019; Vayro et al., 2020), individual time composition appears to be especially important for adherence and might help to dissolve the conflict of prioritizing work over help-seeking reported in other studies (Vayro et al., 2020).

The appreciation of the occupational background as well as the adaptation to it, could be key factors in the coaching conduct. Openly addressing of farming-specific topics and expression of appreciation in the communication about mental health care might therefore be important for actual usage, which are highlighted by studies focusing on help-seeking behavior and intervention design in this population (Lotfi et al., 2010; Vayro et al., 2020). To adapt the actual coaching approach better to the participants' need (Cole and Bondy, 2020; Stumpp and Sauer-Zavala, 2021) knowing and understanding the occupational background is an important skill for the involved coaches. The involvement of experts with farming knowledge was previously recommended by Bowyer et al. (2023) to improve retention in online CBT intervention for farmer's mental health as a conclusion of their acceptability study.

The mentioned supporting aspects of intervention design being adapted to participants' background, therapeutic relationship, anonymity, and easy accessibility as drivers for intervention use were also found in interviews with farmers participating in an online intervention to prevent depression (Freund et al., 2022). However, as expected in the beginning, some of the typical barriers for internet-based interventions reported in farming and non-farming communities such as data security worries, lack of personalization, insufficient computer skills, or internet availability (Braun et al., 2022; Freund et al., 2022; Gerhards et al., 2011; Holst et al., 2017; Mayer et al., 2019) were not mentioned by our interviewees with regard to the telephone coaching. Telephone interventions therefore enrich the possibilities in the realm of remotely delivered health intervention especially for individuals with restricted internet use and in need of a flexible, highly personalized intervention in which they can participate from their familiar environment.

### 4.3 Implication for practice and research

Some direct implications from the interviews for coaching in farmers can be derived. Personalization as a key aspect for satisfaction could be further enhanced by offering additional videoconferencing, which has been accepted and effective for psychotherapy (Berryhill et al., 2019). Videoconferencing could serve those who are missing visual clues during telephone coaching. It should, however, not be forced on those enjoying the visual anonymity as results from a survey study suggest that telephone interventions might overcome fear of being judged by visual appearance and promote disclosure to mental health experts (Lingley-Pottie et al., 2013). The adaptation of intervention content to the farming background as a further mean of personalization should be kept up and enhanced by training coaches in farming-specific topic as already suggested in other qualitative studies on farmers' mental health (Cole and Bondy, 2020; Vayro et al., 2021).

This study results support the ongoing implementation in routine care by stressing that telehealth can play an important role in the promotion of mental health in underserved communities by eliminating travel times, providing anonymity and a low-threshold access to mental health care. However, besides the overall positive attitudes to telephone coaching and the reported advantages, almost half of the participants stated that it would not have been their first choice given alternatives. Personalization of health care should therefore begin with offering different delivery formats (face-to-face, internet- and telephone-based, blended approaches) and settings (group vs. individual programs) and supporting participants in accessing the health care service that best fits their clinical need and individual preference (Smit and Van, 2013; Stumpp and Sauer-Zavala, 2021).

In routine care, clients are counseled by the SVLFG call center about available services and can choose between different face-to-face, telephone-, and internet-based interventions in the preventive setting. However, in the present study it became obvious that participants could not always clearly name their expectations toward the intervention. Introducing standardized assessments of preferences (e.g., on-site, remote), expectations (e.g., amount of guidance, time investment), and personal characteristics (e.g., treatment experience, computer skills) could help call center agents to guide the decision process to what best serves the individual's needs. Besides individual preferences, this process should also take into account the evidence for the interventions in question and current treatment guidelines as well as considering the associated costs of the different delivery methods.

Scalability is one of the main arguments for implementation of telehealth compared to face-to-face services, but different delivery modes are also associated with varying costs and time investment from health care professionals (e.g., one-on-one coaching vs. unguided internet-based intervention) posing a potential bottleneck in care distribution (Berryhill et al., 2019; Nobis et al., 2018). More research is needed to determine how the best match between participant characteristics, health care resources, and health outcome can be achieved and if combinations of different approaches might be beneficial.

### 4.4 Strengths and limitations

While the theory-based approach, the documented standardized steps, and the triangulation with quantitative results in the same population should be highlighted as strengths of this qualitative study, some limitations should be addressed.

This study provides insights in how the different elements of the UTAUT and Evaluation Model contribute to the acceptance of and the satisfaction with the telephone coaching from a farmer's perspective even though the two constructs could not be distinguished clearly from each other. A conjoint code system was built for both constructs after defining deductive cross-theory dimensions, which solved the construct overlap but reduced the specificity of the study. These kinds of changes are to be expected in qualitative research and should help future researchers in preparing their studies (Schreier, 2014).

More adaption of the analysis process was necessary with regard to the structural elements in the Evaluation Model. The interviews yielded no deeper understanding of three of the proposed characteristics and their influence on satisfaction than the model originally suggested and were thus excluded from the analyses. In both routine care and study setting, participants could take part with no extra costs involved and were coached continuously by the same person, thus the theoretical components of ‘Financing’ and ‘Continuity of Care’ probably rather promoted satisfaction than hindering it. In terms of ‘Availability’, most participants were recruited from a study setting (TEC-A). Therefore, availability was dependent on study participation and randomization luck. Only three participants were recruited under routine care conditions (ImplementIT) resulting in a different enrollment process. The coaching began quickly after enrollment and therefore could pose as a supporting factor in both settings. While routine care experience might have been preferable in order to evaluate in a more detail manner on factors like accessibility and availability, in this study, TEC-A participants could be recruited earlier, thus interview results could already be used in the early stages of the implementation process (Glasgow et al., 2003).

For the expectations toward the intervention, it was only feasible to assess them after participants experienced the intervention, thus making reported (fulfilled) expectations prone to hindsight bias (Groß et al., 2017). A priori acceptability studies are warranted for our intervention and target population of farmers (Gunn et al., 2022, Gunn et al., 2021). We cannot foreclose that our sample might be especially selective and highly satisfied or motivated because of convenient and small sampling. Because of the limited number of unsatisfied participants and intervention dropouts, purposefully sampling those might have increased meaning saturation or identified additional themes but could also have screwed representativeness. Because of the individual time frame of the coaching, it could not be avoided that interviews were conducted with different intervals between coaching and interview, possibly changing perception, for example, on achieved changes initiated by coaching which only become noticeable after time (Reio et al., 2017; Smidt et al., 2009).

Overall, high satisfaction and the sampling method also could explain why we did not reach meaning saturation for all identified hindering themes and why HFs were often not supported by many participants in the validation questionnaire. Recruiting more coaching participants might have improved meaning saturation, however, because of the heterogeneity of the intervention and the deductive-inductive analysis approach, achieving full saturation might not be reasonably assumed for our research question (O'Reilly and Parker, 2013). Some HFs were only named rarely, however, the personalized nature of the intervention should be able to address individual treatment barriers during coaching. The two SFs with comparably lower agreement rate (<50 %) in the validation questionnaire were found in the dimension ‘Telephone as the Communication Medium’ which was already rated dividedly in the interviews and were thus in line with the overall results.

Finally, even though we identified the personalization of the intervention as a key factor for the coaching practice, heterogeneity in coaching conduct and adjustment to the individual challenges generalizability of our research results (Stumpp and Sauer-Zavala, 2021). The next step to evidence-based personalized health interventions should be systems for monitoring and evaluating personalization in telehealth as suggested by Aswad and Lessard (2021).

## 5 Conclusion

Theory-based insights were gained on how dimensions of acceptance and satisfactions might influence interviewed farmer's evaluation of a personalized telephone coaching to prevent depression. Perceived changes initiated by coaching, low effort through telephone conduct, and the indicated personalization in terms of time management and farming specificity were perceived key for this overall accepted and satisfactory intervention. While these results support ongoing implementation, more research is needed to optimize personalization and resource input in telehealth care to support sustainable implementation into routine care and reduce depression burden.

## Funding

The German insurance company SVLFG provided a financial contribution to the Friedrich-Alexander-Universität Erlangen-Nürnberg and 10.13039/501100008977Ulm University as expense allowance. SVLFG had no role in study design, data collection, analysis, interpretation and decision to prepare or publish this manuscript. We acknowledge financial support by Deutsche Forschungsgemeinschaft and Friedrich-Alexander-Universität Erlangen-Nürnberg within the funding programme "Open Access Publication Funding"

## Ethical approval

The ethics committee of the Friedrich-Alexander-Universität Erlangen-Nürnberg had approved this qualitative interview study as part of the implementation study ImplementIT and was registered in the German Clinical Trial Registration (DRKS00017078). Interviewees participated in the telephone coaching as clients under routine care conditions during the pilot phase of ImplementIT or as study participants in the associated RCT (TEC-A, trial registration: DRKS00015655). All participants provided extra written informed consent for the interviews, data-matching with RCT data (if applicable), and audio-recording.

## CRediT authorship contribution statement

DDE, HB and MB obtained funding for the SVLFG evaluation project. JT, IT, JF and LB developed the study design and interview guide. JT was responsible for recruitment of interview participants, coordination, collection of interview data, development of code system and analysis. CB and IT provided feedback on the analytically derived code system. IT supervised and contributed to the trial management, data collection and analyses as operational lead of the project and qualitative research expert. JT drafted the manuscript. IT supervised and contributed to the further writing of the manuscript. All authors provided critical revision of the article and approved the final manuscript.

## Declaration of competing interest

IT reports to have received fees for lectures/workshops in the e-mental-health context from training institutes and congresses for psychotherapists. She was the scientific project leader for the research project ImpleMentAll (11/2017 -03/2021, funded by the European Commission) at the Institute for health training online (GET.ON) which aimed to investigate the effectiveness of tailored implementation strategies compared to implementation as usual. DDE has served as a consultant to/on the scientific advisory boards of Sanofi, Novartis, Minddistrict, Lantern, Schoen Kliniken, Ideamed and German health insurance companies (BARMER, Techniker Krankenkasse) and a number of federal chambers for psychotherapy. DDE and MB are current or former stakeholders of the GET.ON Institute for health training online, which aims to implement scientific findings related to digital health interventions into routine care. MB is scientific advisor of mentalis GmbH, a provider for digital aftercare. HB reports to have received consultancy fees, fees for lectures or workshops from chambers of psychotherapists and training institutes for psychotherapists and license fees for an Internet-intervention. JF, CB, JT, LB report no conflicts of interest.

## Data Availability

Access to the anonymized interview transcripts and the interview guide (in German) can be provided to fellow researchers upon request, depending on to be specified data security and data exchange regulations.

## Appendix A Supplementary data

Supplementary material Image 1

## Footnotes

1 MDD: major depression disorder.

2 SVLFG: Social Insurance for Agriculture, Forestry and Horticulture.

3 RCT: randomized controlled trial.

4 ImplementIT: study acronym, Implementation of internet- and tele-based prevention programs.

5 CBT: cognitive behavioral therapy.

6 TEC-A: study acronym, telephone coaching for agriculturists.

7 PHQ: Patient Health Questionnaire.

8 UTAUT: Unified Theory of Acceptance and Use of Technology.

9 QIDS-SR16: Quick Inventory of Depressive Symptomology.

10 CSQ-I: adapted Client Satisfaction Questionnaire for Internet-based Interventions.

11 WAI-SR: Working Alliance Inventory - Short Revised.
