# Supplementary material for: How to promote usage of telehealth interventions for farmers' mental health? A qualitative study on supporting and hindering aspects for acceptance and satisfaction with a personalized telephone coaching for depression prevention
Source: Internet Interv. 2023 Sep 19;34:100671. doi: 10.1016/j.invent.2023.100671 (PMC10523267; doi:10.1016/j.invent.2023.100671)

Online Supplement to:

Thielecke, J., Buntrock, C., Johanna Freund, Braun, L., Ebert, D. D., Berking, M., Baumeister, H., & Titzler, I. (2023). How to promote usage of telehealth interventions for farmers' mental health? A qualitative study on supporting and hindering aspects for acceptance and satisfaction with a personalized telephone coaching for depression prevention

Content:

Supplement 1: COREQ checklist

Supplement 2: Overview over study and analysis procedure

Supplement 3: Overview over all identified themes and their relation to the theoretic components

Supplement 4: Comparison of identified themes in interview and validation questionnaire

Supplement 1: COREQ checklist

Supplement Table 1. COnsolidated criteria for REporting Qualitative research (COREQ): 32-item checklist.

No. Item	Guide questions/description	Reported
Domain 1: research team and reflexivity		
Personal characteristics		
1. Interviewer/-facilitator	Which people conducted the interview or focus group?	Jana Burger (JB)<br>Melanie Martin (MM)
2. Credentials	What were the researcher's credentials? E.g. PhD, MD	Bachelor of science, psychology
3. Occupation	What was their occupation at the time of the study?	Master students
4. Gender	Was the researcher male or female?	Female
5. Experience and training	What experience or training did the researcher have?	Role-play training, feedback, exchange, and supervision by authors
Relationship with participants		
6. Relationship established	Was a relationship established prior to study commencement?	TEC-A RCT participants had already been in contact with the study team prior to the interviews but not to the interviewers. Pilot study participants had no prior contact
7. Participant knowledge of the interviewer	What did the participants know about the researcher? (e.g. personal goals, reasons for doing the research)	The interviewers were not prior known to the participants. Participants were informed in the beginning of the interviews about the background of this study and that it was part of the model project "With us in balance." Research question was described as "interest in positive as well as negative experiences with the coaching"
8. Interviewer characteristics	What characteristics were reported about the interviewer/-facilitator? (e.g. bias, assumptions, reasons, and interests in the research topic)	Interviewers conducted the interviews as part of their master theses and could disclose that to participants
Domain 2: study design		
Theoretical framework		
9. Methodological orientation and theory	What methodological orientation was stated to underpin the study? (e.g. grounded theory, discourse analysis, ethnography, phenomenology, or content analysis)	Inductive-deductive approach (e.g. theory-based analysis and content analysis). The interview guide and interpretation was based on the adapted version of:<br>Theory of Acceptance and Use of Technology (UTAUT) [25]<br>Discrepancy Theory of Satisfaction (Fox & Storms, 1981)<br>Evaluation categories for satisfaction with health care service (Blum, 1998)<br>Quality of Care model (Donadebian, 1968)

Supplement Table 1 (continued). COnsolidated criteria for REporting Qualitative research (COREQ): 32-item checklist.

No. Item	Guide questions/description	Reported
Participant selection		
10. Sampling	How were participants selected? (e.g. purposive, convenience, consecutive, or snowball)	Convenience
11. Method of approach	How were participants approached? (e.g. face-to-face, telephone, mail, or e-mail)	RCT: Via mail through the study team<br>Implementation study: via coaches
12. Sample size	How many participants were in the study?	*N* = 20
13. Non-participation	How many people refused to participate or dropped out? Reasons?	Potentially 66 people were contacted and invited,<br>32 indicated initial interest, 20 signed inform consent, scheduled an interview, and participated
Setting		
14. Setting of data collection	Where was the data collected? (e.g. home, clinic, or workplace)	At home or workplace, via telephone interviews
15. Presence of non-participants	Was anyone else present besides the participants and researchers?	No
16. Description of sample	What are the important characteristics of the sample? (e.g. demographic data, date)	People with an occupational background in farming. Sociodemographic characteristics are reported (see Manuscript Table 2)
Data collection		
17. Interview guide	Were questions, prompts, guides provided by the authors? Was it pilot tested?	Theory-based questions, prompts, and guides were provided. Pilot testing and individual feedback was conducted with the first interview of each interviewer
19. Audio/visual recording	Did the research use audio or visual recording to collect the data?	Interviews were audio-recorded (sipgate & audacity)
20. Field notes	Were field notes made during and/or after the interview or focus group?	No additional notes were made
21. Duration	What was the duration of the interviews or focus group?	Average interview duration was *M* = 44 min (*SD* = 17 min, *Min* = 19 min, *Max* = 78 min)
23. Transcripts returned	Were transcripts returned to participants for comment and/or correction?	No
Domain 3: analysis and findings		
Data analysis		
24. Number of data coders	How many data coders coded the data?	Katja Bouje<br>Vanessa Oth, B.Sc.
25. Description of the coding tree	Did authors provide a description of the coding tree?	Yes
26. Derivation of themes	Were themes identified in advance or derived from the data?	Main categories were identified in advance as part of the dimensions of the theories; themes were derived from the data based on an inductive approach in order to identify what led to support or hindering influence in the dimensions

Supplement Table 1 (continued). COnsolidated criteria for REporting Qualitative research (COREQ): 32-item checklist.

No. Item	Guide questions/description	Reported
27. Software	What software, if applicable, was used to manage the data?	MAXQDA (VERBI software, 2020)
28. Participant checking	Did participants provide feedback on the findings?	Yes, a validation survey was carried out with 15 (75%) of participants responding
Reporting		
29. Quotations presented	Were participant quotations presented to illustrate the themes/findings? Was each quotation identified (e.g. ID)?	Yes, quotations including the ID of the participants are reported
30. Data and findings consistent	Was there consistency between the data presented and the findings?	Yes
31. Clarity of major themes	Were major themes clearly presented in the findings?	Yes, all dimensions and themes are reported (see Figure 2 and Supplement 2) and the most often named are elaborated in detail in the main text
32. Clarity of minor themes	Is there a description of diverse cases or discussion of minor themes?	Yes, diverse cases are discussed

Table based on: Tong A, Sainsbury P, Craig J. Consolidated criteria for reporting qualitative research (COREQ): a 32-item checklist for interviews and focus groups. International Journal for Quality in Health Care. 2007. Volume 19, Number 6: pp. 349–357.

Supplement 2: Overview over study and analysis procedure

Supplement Figure 1. *Visualized data collection and analyses procedure as suggested by Mayring (2015).*

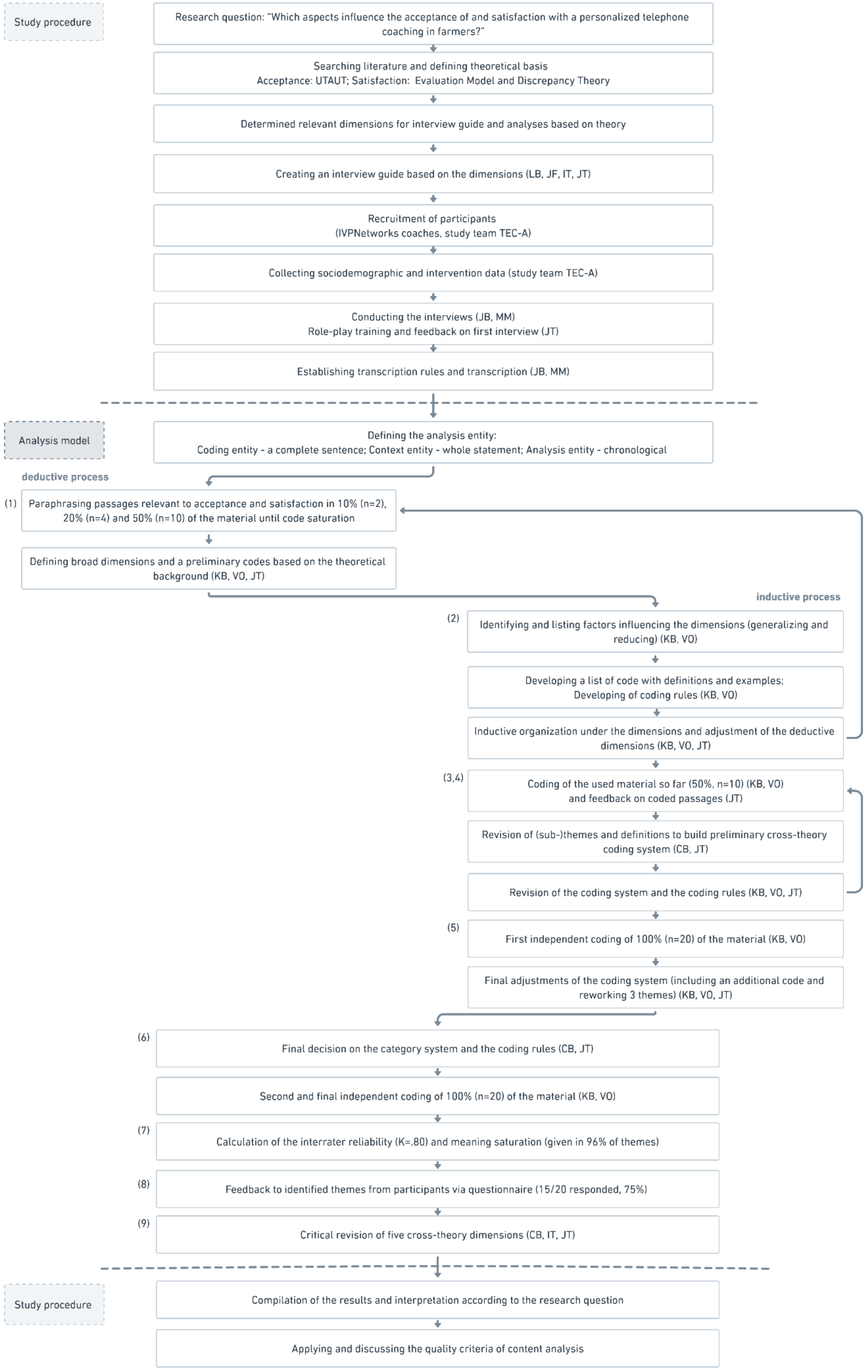

Supplement 3: Overview over all identified themes and their relation to the theoretic components

Figure 1. *Overview on all identified themes with regard to the theoretic foundation of the study.*

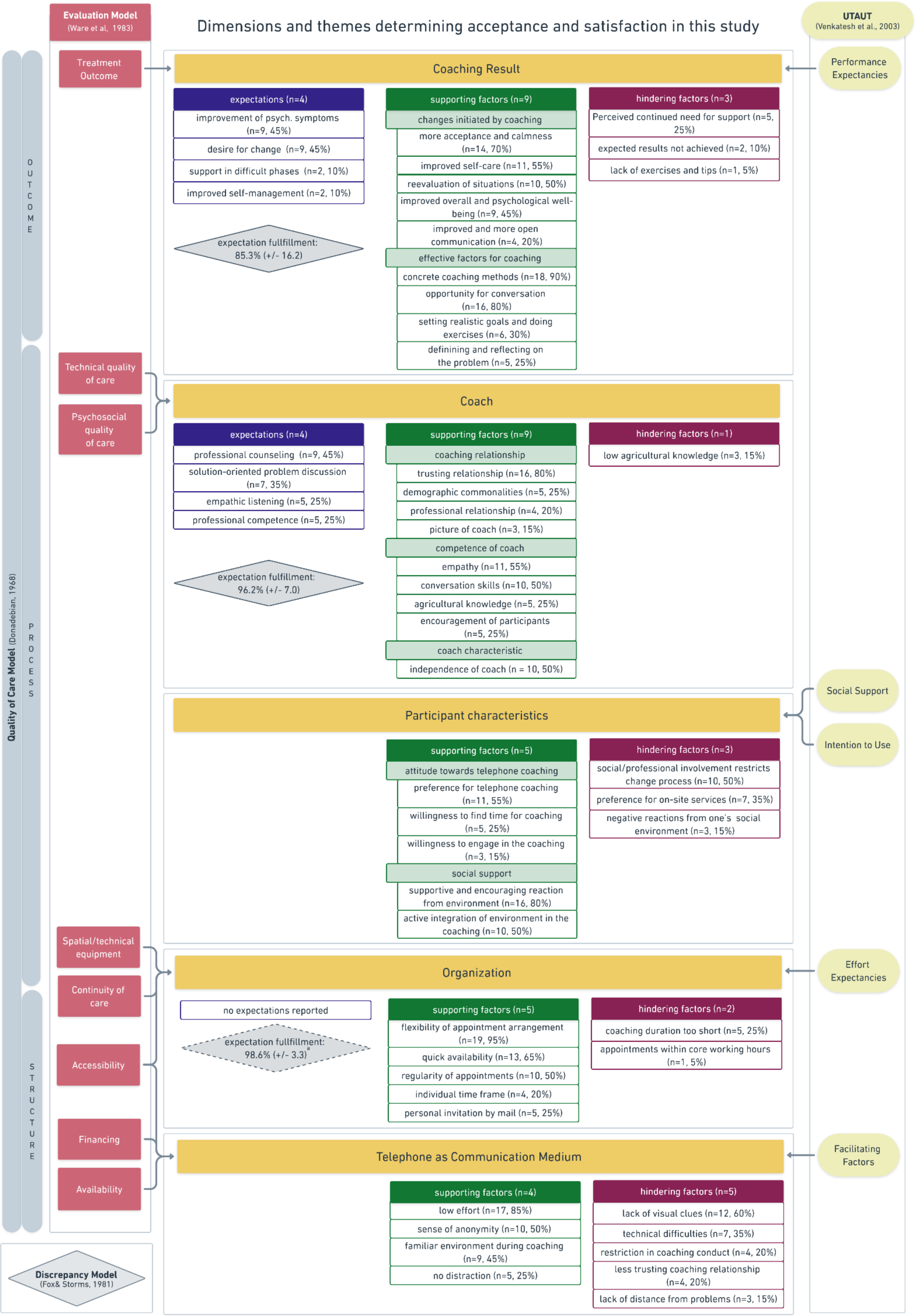

Notes. Arrows indicate which theoretical component is represented in the dimension of the coding system. [a]Participants reported experienced instead of expected organization. Expectation fulfillment rating can be interpreted as degree of satisfaction with the addressed organization.

Supplement 4: Comparison of identified themes in interview and validation questionnaire

Supplement Figure 2. Frequency of identified themes in the interviews (n=20) and the validation questionnaire (n=15) influencing acceptance of and satisfaction with the telephone coaching.

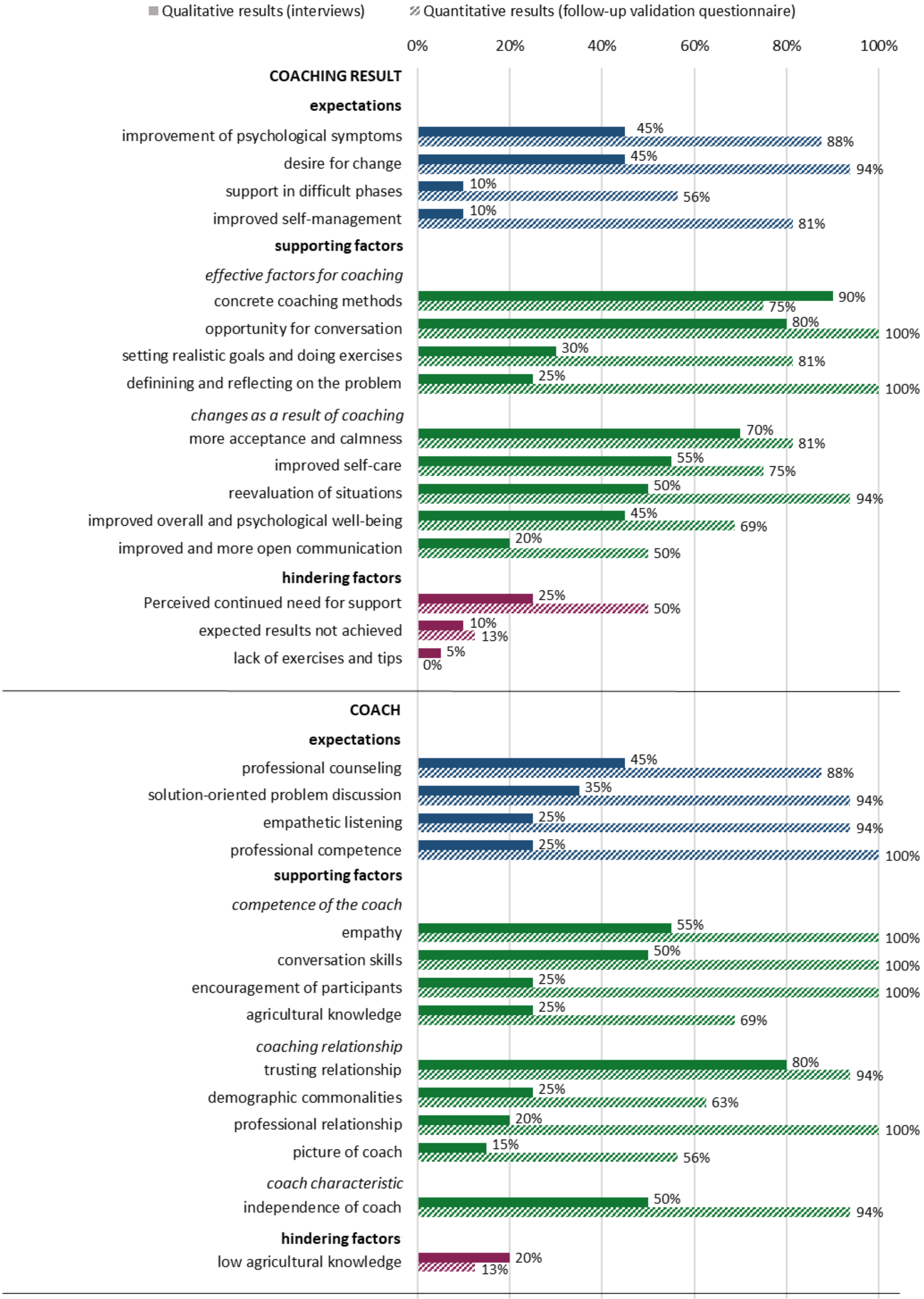

Supplement Figure 2 (continued). Frequency of identified themes in the interviews (n=20) and the validation questionnaire (n=15) influencing acceptance of and satisfaction with the telephone coaching.

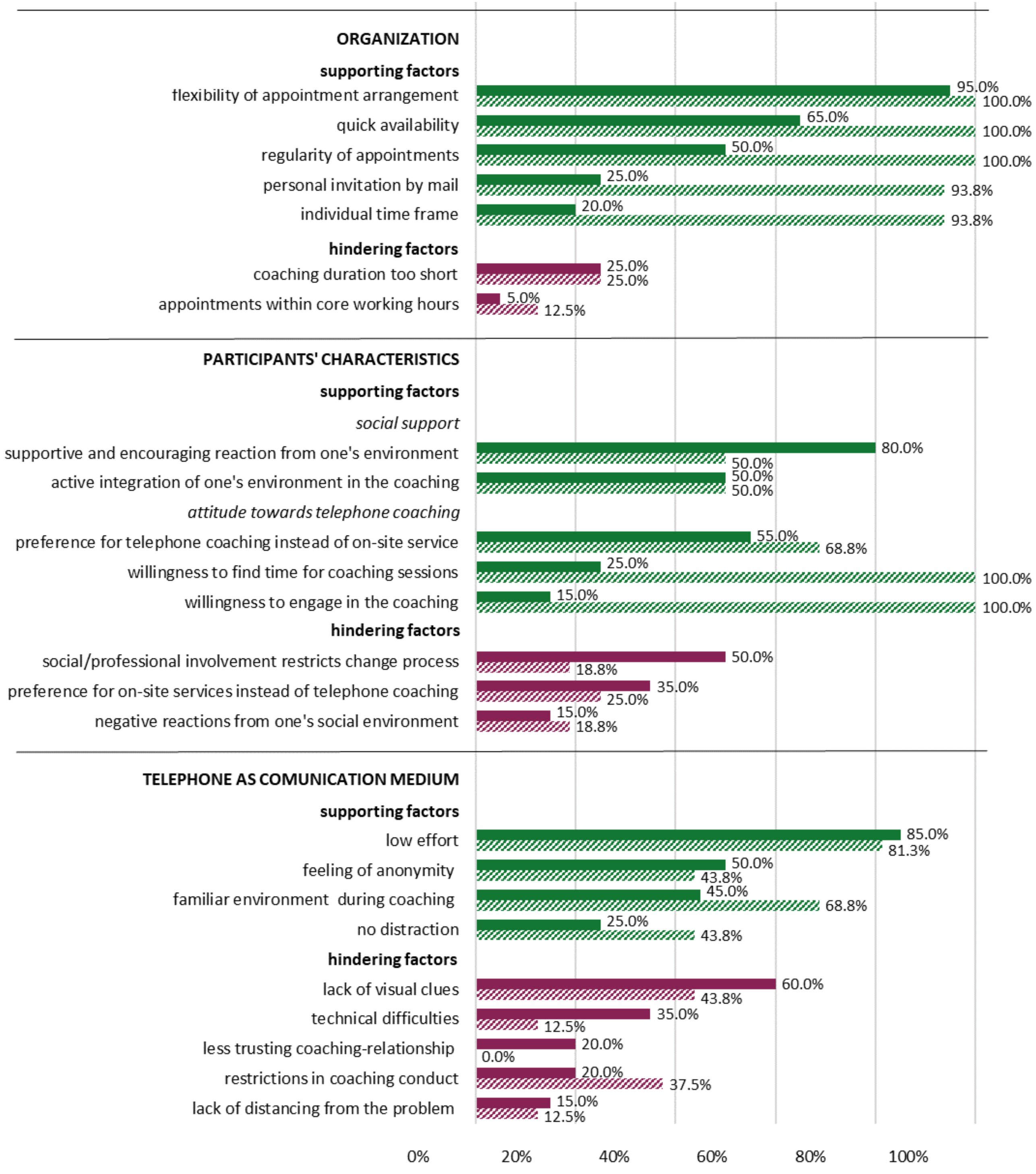

Supplement: Supplementary file 1 — Supplementary material [file mmc1.pdf]
